# Social norms and security and justice services for gender-based violence survivors in Nepal: Programmatic implications from a mixed-methods assessment

**DOI:** 10.1371/journal.pone.0297426

**Published:** 2025-10-16

**Authors:** Cari Jo Clark, Brian Batayeh, Iris Shao, Irina Bergenfeld, Manoj Pandey, Sudhindra Sharma, Shikha Shrestha, Amritha Gourisankar, Anudeeta Gautam, Tehnyat J. Sohail, Holly Shakya, Grace Morrow, Abbie Shervinskie, Subada Soti

**Affiliations:** 1 Emory University, Rollins School of Public Health, Hubert Department of Global Health, Atlanta, Georgia, United States of America; 2 Emory University, Rollins School of Public Health, Department of Epidemiology, Atlanta, Georgia, United States of America; 3 Volunteer Service Overseas International, Kathmandu, Nepal; 4 Interdisciplinary Analysts, Kathmandu, Nepal; 5 Emory University School of Medicine, Atlanta, Georgia, United States of America; 6 Lewis Katz School of Medicine at Temple University, Philadelphia, Pennsylvania, United States of America; 7 Georgia State University, Atlanta, Georgia, United States of America; 8 University of California San Diego, Department of Medicine, Division of Global Public Health, San Diego, California, United States of America; Northeastern University, UNITED STATES OF AMERICA

## Abstract

**Background:**

Gender-based violence (GBV) is highly prevalent throughout the world. Only a small fraction of survivors seek help from security and justice (S&J) providers such as the police or courts, due in part to social norms that discourage help-seeking. The prevention of GBV requires attention to both demand- and supply-side factors and programming is moving toward this integration, including in Nepal. However, little research exists at the nexus of these issues. To address this gap, we provide a comprehensive mixed-methods situation analysis of GBV-related social norms, help-seeking, and S&J service provision.

**Methods:**

Data included a household survey (N = 3830), a sub-study of youth (N = 143) and married adults (N = 464) in one site and qualitative data collection including interviews with S&J service providers, help-seeking GBV survivors and families (N = 68), and focus group discussions with police, youth groups, and school management committees (N = 20) in four sites. Descriptive analysis of survey data was triangulated with findings from a modified grounded theory analysis of the qualitative data to elucidate the role of social norms and other barriers limiting help-seeking.

**Results:**

GBV was perceived to be common, especially child marriage, domestic violence, eve-teasing, and dowry-related violence. Formal help-seeking was low, despite positive attitudes towards S&J providers. Participants described injunctive norms discouraging formal reporting in cases of GBV and sanctions for women violating these norms.

**Conclusions:**

Norms favoring family- and community-based mediation remain strong. Sanctions for formal reporting remain a deterrent to help-seeking. Leveraging gender-equitable role models, such as female S&J providers, and connecting S&J providers to women and youth may capitalize on existing shifts.

## Introduction

Gender-based violence (GBV) is any harmful act directed at an individual or group based on their gender. GBV includes, but is not limited to, intimate partner violence (IPV), in-law abuse, sexual assault, dowry-related violence, child marriage, and sexual harassment or assault, with IPV being the most pervasive. Globally, approximately 1 in 3 women have experienced physical or sexual IPV [1], but less than 9% of survivors have sought help from the police [2], a prominent actor in the delivery of services meant to safeguard personal safety and security and uphold the rule of law. This is despite decades of legal and procedural improvements: currently 85% of the 190 countries surveyed by the World Bank’s Women, Business, and the Law project, have criminalized domestic violence [3]. Over this time, much has been accomplished, including a shift toward survivor-centered, gender sensitive services, improved legal proceedings, and outcome enforcement. However, these improvements are modest relative to the investment, in part due to social norms that discourage disclosure outside the family and the interference of others in private affairs.

The prevention of GBV requires attention to both demand- and supply-side factors and programming is moving toward this integration, including in Nepal. However, little research exists globally at the nexus of these issues. To address this gap, **we provide a comprehensive mixed-methods situation analysis of GBV-related social norms, help-seeking, and security and justice (S&J) service provision in the Madhesh and Lumbini provinces in Nepal.**

## Background

### GBV in Nepal

In Nepal, an estimated 23% of reproductive age women report having experienced physical violence victimization since age 15 and 8% report sexual violence victimization in their lifetime, with the Terai regions (i.e., lowlands), such as areas in the Madhesh and Lumbini provinces, having the highest prevalence estimates compared to hilly or mountainous regions [4]. Roughly 32% and 16% of ever married/partnered women aged 15–49 years in Madhesh and Lumbini provinces, respectively, experienced IPV, a common form of GBV, in the last 12 months [4]. When considering traditional practices such as *chhaupadi* (isolation of women and girls during menstruation and just after childbirth) and child marriage, the GBV prevalence estimates would exceed 80% in areas where these practices are endemic. Despite progress over the past few decades, such as making IPV, *chhaupadi*, child marriage, and dowry punishable offenses, raising the legal marriage age to 20 years, increasing the inclusion of women in the political realm, and supporting increased education for girls, women in Nepal often experience limitations in their ability to seek education, employment, or socialize freely outside the home. Women remain less educated than men, with 46% of women aged 15–49 years in Madhesh province and 22% in Lumbini province having no formal education compared to 19% and 7% of men in the respective provinces. Furthermore, while 76% and 80% of men aged 15–49 years in Madhesh and Lumbini, respectively, are employed, only 50% and 60% of women hold employment [4]. Nepali men tend to be dominant in household decision-making as only 39% and 46% of married women in Madhesh and Lumbini, respectively, participate in decisions in all of the following categories: their own healthcare, major household purchases, and visits to their family [4]. Finally, men often ascribe to traditional gender roles and hold significant control over their wives. The acceptability of IPV and other forms of GBV remains relatively high among Nepali men and women, maintaining the vulnerability of women and girls [4–6].

### S&J sector in Nepal

The S&J sector in Nepal is a mixture of formal and informal services. Indigenous practices of mediation or adjudication by local elders and leaders, the oldest of these informal services, are easily accessible, efficient, and a means to preserve family honor and social harmony. However, these practices are male-dominated and prioritize social harmony over individual needs, i.e., they are not survivor-centered and may be politicized, especially when justice is facilitated by persons affiliated with a political party. Formal S&J actors (e.g., police, court system, lawyers) are a more recent development, but there have been setbacks in establishing their utility, including accusations of political interference; corruption; discrimination; physical, financial, and social inaccessibility; and a lack of timely resolution, especially for the judiciary [7–13]. Despite these challenges, considerable reforms in infrastructure, equipment, and professionalism are underway. Importantly, these reforms have begun among the police who have become a preferred source of assistance for crime, especially for domestic violence cases in the Madhesh and Lumbini provinces where the present study is set. A special directorate of the police was established to address a lack of professional skills within the police force when addressing GBV and related crimes against women, children, and elders, including gender responsive and victim centered approaches. While well-regarded, its reach into the community remains less than that of the traditional police force. After the 2017 elections, Judicial Committees were established to bring judicial services closer to the community as part of the decentralization process. These committees are headed by the deputy mayor of urban municipalities and the vice-chairperson of rural municipalities, newly formed posts through the government restructuring process headed predominantly by women—an important opportunity to support female leadership development. The committees, while established throughout the country, have been criticized for insufficient legal training to effectively address GBV and are not yet fully resourced to perform their mandate. This legal capacity deficit is not unique to the Judicial Committees but a broader challenge arising from the devolution of governance to the local level.

### S&J Help-seeking for GBV

In Nepal, as in many settings around the world, a general lack of help-seeking for GBV predominates [14]. According to nationally representative data of reproductive age women, only 8.7% of women who had experienced physical or sexual violence sought help from formal sources in 2022, with the police being the most common (7.2%). Instead, these women more often turned to informal sources, such as their maternal family (63%) and neighbors (35%) to help end the violence [4]. Non-disclosure has been linked to restrictive gender norms, risk of further abuse or social repercussions, financial dependency, a lack of knowledge of services, limited faith in the justice system, and preference for community-based mediation to maintain social harmony; further, inaccessible, under-resourced, gender insensitive, and trauma uninformed services limit help-seeking from the supply side [9,15–22]. Similarly, poor and marginalized communities do not seek police and justice support, as they perceive services as discriminatory based on ethnicity, caste, income level, and gender [10].

## Materials and methods

### Overview

This study is a concurrent mixed-methods situation analysis of GBV-related social norms, help-seeking, S&J service provision conducted between 14-08-2019 and 07-09-2019 in the Madhesh and Lumbini provinces of Nepal. The impetus for the study was Strengthening Access to Holistic, Gender Responsive, and Accountable Justice (SAHAJ), a project developed and implemented by a consortium of international and local non-governmental organizations lead by VSO Nepal, in which the authors of the study were embedded at the time of the study. The project was one of a number of projects in a larger initiative, the Integrated Programme for Strengthening Security and Justice (IP-SSJ) in Nepal, which was funded by the Department for International Development (DfID, currently known as the Foreign Commonwealth and Development Office of the United Kingdom) and executed by the Ministry of Women and Social Welfare and the Ministry of Home Affairs. The overall objective was to improve access to modernized, gender-sensitive, and disaster resilient police units and increase knowledge of the law and services available to prevent and respond to violence against women and children. The SAHAJ project itself was designed to reduce GBV by breaking the culture of silence and increasing access to survivor-centered S&J services using a multi-component S&J and social norms intervention taking a community approach and targeting families, schools, survivors, and S&J providers. The social norms programming included: 1) a family-centered approach involving gender transformative workshops for family members and income generating activities for women; 2) a school-centered approach involving enhanced response and referral among school management, the development of youth clubs to advocate for norms transformation, and the establishment of linkages between students and GBV service providers; and 3) intergenerational dialogues and leader engagement in norms-focused events at the community level [23]. The S&J component included verdict monitoring, strengthening referral pathways, training service providers in gender, norms and survivor-centered services, and enhancing police – community accountability and partnership through a community score card approach [24,25]. The ultimate goal of the study was to assess the impact of layering on programmatic impact, with one set of study sites containing all programmatic elements including S&J and social norms programming targeting families and schools, one set of sites containing only S&J programming, and a final set of sites containing only family- and school-based programming. Sites with combined programming were hypothesized to demonstrate greater improvement in norms and confidence in S&J providers at study endline. Given the persistent lack of information at the intersection of social norms and S&J interventions globally, this study begins to address this gap by providing a detailed assessment of GBV-related social norms, help-seeking, and S&J service provision in 17 sites across nine out of the 13 SAHAJ project districts in Madhesh and Lumbini provinces in Nepal.

### Site selection

**Fig 1:**
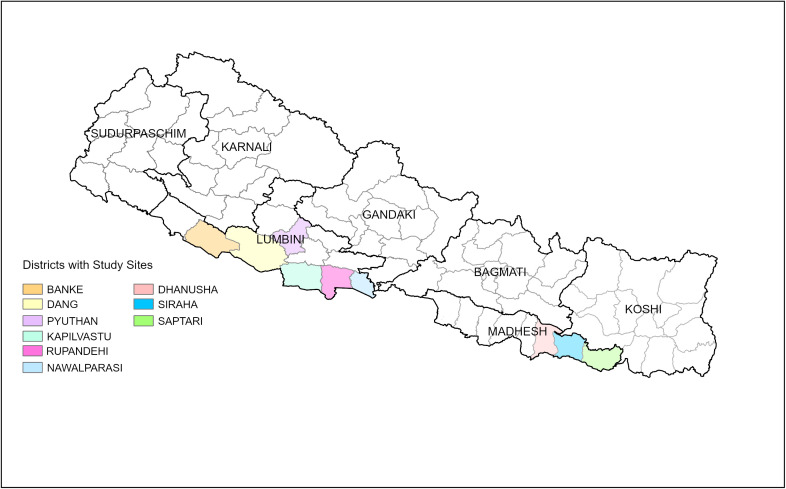
Map of Nepal provinces and districts with study sites. Data by GeoBoundaries provided under the Creative Commons Attribution 4.0 International (CC BY 4.0) license (https://www.geoboundaries.org/) Licensed Under Creative Commons Attribution 4.0 International (CC BY 4.0) (https://creativecommons.org/licenses/by/4.0/).

SAHAJ project sites were chosen in negotiation with the government of Nepal to build upon existing activities under the IP-SSJ project. The different components of the SAHAJ intervention were conducted on different geographic scales within the municipalities. The family- and school-based programming occurred in toles (the smallest geographic unit, formerly the smallest administrative unit) and the S&J-focused activities were designed to impact the population across toles in wards (the current smallest administrative unit which is an aggregate of toles). Site selection was purposive in project sites in which the family- and school-based programming was administered and involved discussions between the intervention-implementing organizations and local government, police, non-governmental organizations, and committees tasked with GBV prevention and response to identify communities in particular need of programming that also lacked existing social norms programming. In project sites in which S&J programming was administered, wards were segmented into toles, and one tole was randomly selected for the study. In addition, the research team selected one tole in one municipality in the Lumbini province to serve as the site of a more in-depth sub-study, intentionally chosen based on the implementing partners’ perceived high rates of child marriage, polygamy, the practice of dowry, domestic violence, and mobility restrictions for women, although specific statistics were not available at this geographic level. Within two sites in each province, the qualitative portion of the study was administered with the goal of maximizing variation across the sites [26]. Within two sites in each province, the qualitative portion of the study was administered with the goal of maximizing variation across the sites [26]. Fig 1 below shows the provinces and districts of Nepal in which study activities took place.

### Sampling and eligibility

We conducted a household survey in each study site. The household listing included approximately 225 households in each site. Sites that were larger than this size were segmented and those that were smaller were combined with adjacent toles to obtain the required sample size. Within each household, a married woman 18 years or older, or if not available, any knowledgeable adult 18 years or older (N = 3830; 99.7% female) was administered the household survey. Married women were targeted as they would more likely be at home compared to men and we wanted to identify the most consistent respondent type possible to remove some variability that would be introduced by having a much larger range of respondents. For the sub-study within the Lumbini Province, we additionally surveyed all adolescents aged 15–19 or in grades 9–12 (male: N = 75; female: N = 68) and married adults (men: N = 221; women: N = 243). Respondent characteristics are presented in **Table 1**. Key differences across the respondent groups reflect the sampling strategy and include the predominance of women respondents in the household survey compared to a more equal representation of male and female respondents in the sub-study; younger age and better education among the youth sample compared to the other samples, and the predominance of religious minorities in the sub-study, which reflects the socio-demographic characteristic of the district which proportionally contains more Muslims than other districts. Employment status was only assessed for adult men and women in the sub-study, and though roughly half of these respondents were employed, only 21% of women held employment compared to 88% of men. There were limited differences between the respondents from Madhesh province and Lumbini province aside from a lack of rural municipalities in Madhesh compared to equal representation of urban and rural respondents in Lumbini, and a higher percentage of persons of upper caste in Lumbini compared to Madhesh.

**Table 1:** Sample size and socio-demographics, by survey type.

	Household survey			Sub-study	
	Total (N = 3830)	Madhesh (N = 1577)	Lumbini (N = 2253)	Adult survey (N = 464)	Youth survey (N = 143)
**Province, %**					
Madhesh	41.2	100.0	–	–	–
Lumbini	58.8	–	100.0	100.0	100.0
**District**, %					
Siraha	5.9	14.3	–	–	–
Saptari	29.3	71.1	–	–	–
Dhanusha	6.0	14.5	–	–	–
Kapilvastu	23.5	–	40.0	100.0	100.0
Rupandehi	11.7	–	20.0	–	–
Nawalparasi	5.8	–	9.9	–	–
Dang	5.9	–	10.0	–	–
Pyuthan	6.0	–	10.1	–	–
Banke	5.9	–	10.0	–	–
Urban municipality, %	58.7	100.0	49.9	–	–
Rural municipality, %	41.3	0.0	50.1	–	–
**Gender**, %					
Male	0.3	0.2	0.3	47.6	52.5
Female	99.7	99.8	99.7	52.4	47.6
**Age,** (M, SD)	38.6 (13.3)	37.4 (12.4)	39.5 (13.8)	40.4 (13.1)	17.0 (2.0)
**Caste**, %					
Dalit	24.2	35.4	16.3	7.5	6.3
Disadvantaged Janajatis	6.5	10.7	3.6	1.7	4.2
Disadvantaged Non-Dalit Terai	29.9	33.0	27.7	7.8	7.0
Religious Minorities	15.8	9.6	20.1	59.1	61.5
Relatively Advantaged Janajatis	1.6	2.0	1.3	0.9	0.0
Upper Caste	21.6	8.6	30.7	22.6	19.6
Others	0.5	0.6	0.4	0.4	1.4
**Highest Grade,** (M, SD)	2.5 (3.9)	2.3 (3.8)	2.6 (4.0)	5.7 (4.7)	8.6 (2.6)
**Employed, %**					
Yes	–	–	–	52.6	–
No	–	–	–	47.4	–

Note: Decimals may not add to 100 due to rounding.

In the four sites identified for qualitative data collection (**Table 2**), we held focus group discussions (FGDs) with adolescents in youth clubs (N = 8 groups, 70 individuals), ward police (N = 4 groups, 33 individuals), members of the school management committee (N = 4 groups, 28 individuals) and the women’s police cell (N = 4 groups, 24 individuals). We also conducted in-depth interviews (IDIs) with formal and informal S&J providers (police chief, women’s police cell, GBV control group member, and local government officials including a judicial committee representative; N = 19), GBV help-seeking survivors (N = 4), school administrators (N = 4), and 12 families (N = 41 individuals) recruited into SAHAJ family-based programming. The husband, wife, mother-in-law, and father-in-law of each family in the Madhesh province were interviewed, and in the Lumbini province, the mother, father, and adolescent daughter of each family were interviewed. This slight difference in family members reflected the focus of each province, which was married women’s safety and help-seeking in the Madhesh province and adolescent girls’ safety and help-seeking in the Lumbini province. **Table 2** summarizes the qualitative data collected within each site.

**Table 2:** Qualitative sample, by data collection mode, S&J provider type, and location.

Data collection format	Stakeholder type	District (Municipality)			
		Siraha^*^ (Siraha)	Saptari^*^ (Rajbiraj)	Kapilvastu^†^ (Kapilvastu)	Rupandehi^†^ (Marchwari)
FGD Participants	Youth group female	7	8	10	11
	Youth group male	7	8	10	9
	School management committee	9	9	5	5
	Ward police	8	11	7	7
	Officers in the women’s cell	6	6	6	6
IDI Participants	Police chief	1	1	1	1
	Officer in the women’s cell	1	1	1	1
	GBV Control Group member	1	1	1	1
	Judicial Committee member	2	2	1	2
	Help-seeking survivor	1	1	1	1
	School administrator	1	1	1	1
	Husband	3	3	2	3
	Wife	3	3	3	3
	Wife’s mother-in-law	3	3		
	Wife’s father-in-law	3	3		
	Adolescent girl			3	3

Note: FGD = focus group discussions; GBV = gender-based violence; IDI = in-depth interviews; ^*^Madhesh Province; ^†^Lumbini Province

### Data

Data collected was complementary across qualitative and quantitative data collection tools (**Table 3**).

**Table 3:** Measurement topics and respondents by data collection tool.

Topic	Household survey	Sub-study survey	Sub-study survey	In-depth interview	In-depth interview	In-depth interview	Focus group discussion
	(Married adult women or other)	Adolescents aged 15–19	Married adults	S&J providers	Help-seeking survivor	School administrators and families	Adolescents, school management committee, ward police, women’s police cell
Descriptive norms	X	X	X			X	X
Injunctive norms	X	X	X			X	X
Perceptions about S&J actors	X	X	X				
Perceptions about S&J actors in-depth		X	X				
Women’s exposure to IPV			X (women)				
Women’s exposure to in-law abuse			X (women)				
Child maltreatment		X					
Service context				X			
Help-seeking for violence			X (women)		X	X	X
Acceptability of violence and help- seeking							X

Note: S&J = security & justice, IPV = intimate partner violence.

Descriptive norms (perceptions of prevailing practices in the community) were measured in the household survey and the sub-study survey with items developed for the study which assessed whether seven common forms of GBV in Nepal (domestic violence against women, sexual assault of women and girls, dowry related violence, eve-teasing, *chhaupadi,* child marriage, male-to-male violence) were widespread in their community. For the household survey, the respondent was asked whether the statement was true or false. In the sub-study, the participant was asked to rate their level of agreement on a 4-point Likert scale from strongly agree to strongly disagree. The items were dichotomized to obtain a percent agreeing or strongly agreeing versus disagreeing or strongly disagreeing that the practice was prevalent in their community. Injunctive norms (perceptions about what are acceptable practices in the community) were measured in the household survey and the sub-study with 15 items based on the Partner Violence Norms scale [27] and an ongoing IP-SSJ evaluation of the consortium. The respondent was asked to report the extent to which members of their community espoused the sentiment on a 5-point Likert scale from nearly all members of the community to no members of the community. Items were dichotomized representing perceptions that most or nearly all persons in a community held that belief.

Physical and/or sexual IPV was measured among adult women in the sub-study with eight items from the Demographic and Health Survey, which were also measured in the IP-SSJ evaluation. Items measured the frequency (never, sometimes, or often) of the act in the prior 12 months. A dichotomous measure was created representing exposure to any act of IPV. In-law abuse was measured among adult women in the sub-study with two items from a scale developed in prior research in Nepal assessing exposure (yes/no) to physical violence perpetrated by the husband’s family and in-laws’ incitement of partner violence. A dichotomous measure was created representing exposure to either act. Child maltreatment was measured among youth in the sub-study with the 7-item Short Child Maltreatment Questionnaire developed by the World Health Organization Regional Office for Europe [28]. Each item assessed whether the respondent experienced the act never, once or twice, or many times in the past 12 months by an adult in their household. A dichotomous measure was created representing exposure to any act. To assess help-seeking, women reporting violence were asked whether they had told anyone about the violence in the past 12 months. Item response options included 17 types of informal and formal help-seeking choices and no one. Assessment of help-seeking among youth also was planned; however, due to an error in the survey skip logic, we were unable to accurately assess the variable.

Perceptions about S&J actors were measured in the household survey and sub-study with two items from the IP-SSJ monitoring tool which was being used across projects in the consortium to assess whether the police are trustworthy people and whether citizens have a role to play in supporting the police to maintain law and order. Each item was assessed on a 4-point Likert scale from strongly agree to strongly disagree in addition to a “don’t know” response option. Items were dichotomized to obtain a percent agreeing or strongly agreeing versus disagreeing or strongly disagreeing. In the sub-study, participants were asked to respond to six additional questions developed for the study about each of five S&J actors (police, Judicial Committee, Ward Chairperson, local leader, and women’s committee) to assess their perceptions of aspects of victim-centered services in response to a report of physical or sexual violence to that actor. Respondents were asked whether they would be willing to report the case to that actor, whether they believed the case would be taken seriously, whether the survivor would be treated with respect, whether her privacy would be protected, whether the perpetrator would be punished, and whether the survivor would experience negative social repercussions for reporting. Each question was assessed on a 4-point Likert scale from strongly agree to strongly disagree with a response option for “don’t know.” Items were categorized into 1) agree or strongly agree, 2) disagree or strongly disagree, 3) and don’t know.

IDI guides for S&J providers and help-seeking survivors were developed to examine the S&J service context and help-seeking with questions modeled after constructs derived from Social Development Direct’s Security Sector Module. IDIs with families examined decision-making, descriptive and injunctive norms on violence against women and girls and help-seeking for GBV, experience of violence and help-seeking, and engagement in local violence-related committees, with questions developed for the study and vignettes modeled after the Social Norms Analysis Plot (SNAP) framework [29]. We designed FGDs to assess norms, norms’ reference groups or those to whom an individual looks to determine acceptable behaviors, sanctions, sensitivity to sanctions with particular reference to family privacy, and to understand the acceptability of GBV and help-seeking for GBV, with questions developed for the study including vignettes also modeled after the SNAP framework [29,30]. We designed FGDs to assess norms, norms’ reference groups or those to whom an individual looks to determine acceptable behaviors, sanctions, sensitivity to sanctions with particular reference to family privacy, and to understand the acceptability of GBV and help-seeking for GBV, with questions developed for the study including vignettes also modeled after the SNAP framework [30].

### Analysis

Descriptive analyses (frequencies, means, minimums, and maximums) of the household survey data were performed by site and province. For the sub-study, descriptive analyses were conducted by respondent type (adult man, adult woman, male adolescent and female adolescent). For qualitative data, we developed a codebook based on an initial reading of 15 transcripts and added codes over the initial stage of the analysis to incorporate emergent themes. The revised codebook was then used in two rounds of inter-coder reliability testing (evaluated using Cohen’s kappa [31]) among two team members using a subset of the transcripts. Following each round of testing, team debriefs were used to resolve discrepancies and make minor edits to codes and definitions. The team then coded the remaining transcripts. All coding, cross-classification, and inter-coder reliability testing was performed in MAXQDA 18 (Berlin, Germany). A narrative analysis of the family interviews began with a matrix summarizing key social norms and S&J themes in each interview, which was used to create summaries of each family. Thick descriptions using the entire data set were also generated with an analytic focus on key themes, interconnectedness, and synergies.

### Ethics

The study was approved by the Nepal Health Research Council (#602/2019) and the Institutional Review Board at Emory University (IRB00110703). Data collectors participated in a week-long training which focused on topics such as gender-based violence and social norms, ethically collecting sensitive information, building rapport, and nonjudgmental interviewing. To limit bias due to social desirability, less sensitive questions, which made up most of the tools, were placed near the beginning of each tool to aid rapport before asking more sensitive questions. All participants provided written informed consent, signing with an X if non-literate and co-signed by the interviewer. Interviews took place in a private space away from other members of the household and were conducted by a data collector of the same gender as the participant to increase the comfort of the participant. Following ethical guidelines, only one adult woman per household in the sub-study was administered the IPV module to do decrease the risk women experiencing revictimization [32]. Qualitative participants also provided consent to be audio recorded. All data were stored on a secured drive and deidentified prior to analyses.

## Results

### GBV is perceived to be widespread

**Fig 2:**
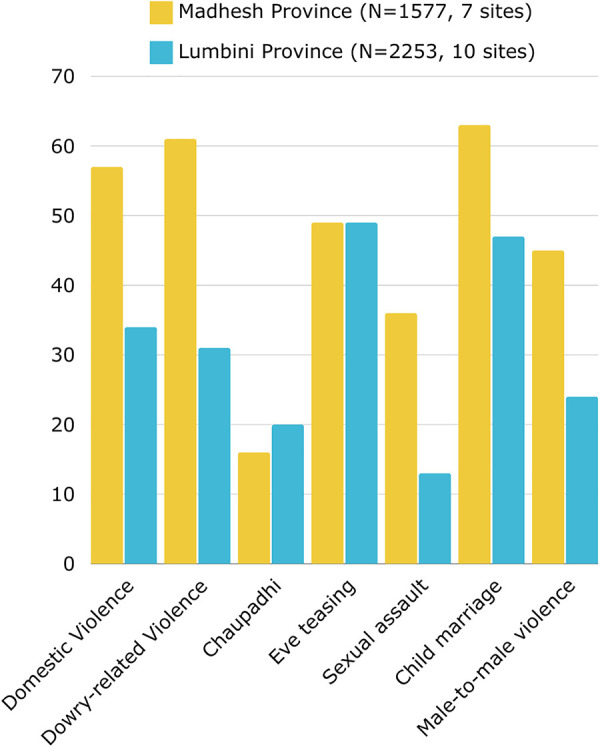
Mean percent agreement with descriptive norms items, by province, household survey.

Quantitatively, descriptive norms suggest that GBV is perceived to be widespread with some regional and locality differences. Forms of violence most commonly reported by participants across the sample included child marriage, eve-teasing (i.e., public sexual harassment or assault), domestic violence, and dowry-related violence. Overall, a greater proportion of individuals in the Madhesh province reported various forms of GBV to be widespread compared to the Lumbini province; however, eve-teasing was reported to be widespread in both provinces, and *chhaupadi* was reported to be widespread by 16% of the sample in the Madhesh Province and 20% of the sample in the Lumbini Province (Fig 2). The tole minimums and maximums suggest considerable diversity in these perceptions within the provinces (S1 Table).

Reports from the qualitative data also revealed that respondents characterized the acts mentioned above, as well as other acts, as forms of GBV. IPV and abuse from in-laws were commonly identified as forms of violence across all four sites and took the form of beatings, verbal abuse, financial abuse (e.g., withholding of basic needs), severe restrictions on mobility, and refusal to provide necessary documents (e.g., birth and marriage certificate). Dowry issues were discussed both as a cause of GBV and a form of GBV and were implicated in both IPV and abuse from in-laws across all sites.

*“When a daughter is married to someone after paying a dowry of 10 lakh and a motorbike, but still after reaching her in-laws house, she will be tortured for not bringing more dowries. Everyone in the family will torture her. They will rebuke her for not bringing more dowries and she will be abused by everyone… Dowry is the major reason for abusing someone.” (Saptari, S&J provider)*

Child marriage also was also widely perceived as prevalent among qualitative respondents. Respondents partially attributed early marriage to low levels of education and awareness, financial strain on the household, reduced dowry for young brides, and concerns about the family’s reputation. According to male youth, girls who are perceived to have relationships with boys or who engage in other “bad” behavior are at risk of early marriage. Child marriage was frequently reported to be connected to broader discrimination against women and girls, which was perceived to be common across sites and often takes the form of denying girls the same educational opportunities as boys. Qualitative participants of all types stated that girls are withdrawn from school earlier, sent to lower quality schools, burdened by household chores, face menstrual restrictions, and are provided with less nutrition while boys study or play.

### Prevalence of GBV differs by type, but common across types is a lack of help-seeking

Based on findings from the survey, the most frequent form of violence reported was child marriage. Among married female survey respondents, 27.3% married before age 15 and 60.2% married before age 18. Help-seeking for child marriage was not assessed as the timing of the experience was not assessed. In the sub-study survey, among the adult female respondents, 4.5% (n = 11) reported physical and/or sexual IPV in the past 12 months (**Table 4**). Approximately 7% (n = 16) of the adult female respondents reported being hit, kicked, punched or otherwise physically hurt by a member of their husbands’ families in the past 12 months and 5.4% (n = 13) reported that their in-laws encouraged their husband to do the same. Of the 9.5% (n = 23) of women who reported IPV or in-law abuse, 91.3% (n = 21) told no one; the remaining two women told family or friends. Among the youth, 14.7% (n = 11) of male respondents and 16.2% (n = 11) of the female respondents reported child maltreatment from a parent or caretaker in the prior 12 months.

**Table 4:** Prevalence of forms of gender-based violence by respondent group, sub-sample.

	Adult women (N = 243)	Female youth (N = 68)	Male youth (N = 75)
Married before age 15, %	16.1	–	–
Married before age 18, %	44.4	–	–
Physical and/or sexual IPV last 12 months, %	4.5	–	–
In-law physical abuse last 12 months, %	6.6	–	–
In-law encouraged abuse last 12 months, %	5.4	–	–
Child maltreatment last 12 months, %	–	16.2	14.7

### S&J providers, particularly police, are an acceptable source of help

Most (87%) of the household survey sample and between 80% and 89% of the sub-study sample (**Table 5**) agreed that police are trustworthy Further, across respondent types, two-thirds or more of sub-study participants reported willingness to report physical or sexual victimization to an S&J provider, believed the case would be taken seriously, the survivor would be treated with respect, the survivor’s privacy would be protected, and the perpetrator would be punished by S&J actors. While this suggests rather broad endorsement of the various S&J providers, **Table 5** also signals differences in perceptions across S&J providers. The highest percentage of survey respondents reported willingness to report the violence to the police (86%). Similarly, the highest percentage of respondents thought the perpetrator would be punished by the police (77%) while the local leader received the fewest endorsements that they would punish the perpetrator (66%). Across all the other survivor-centered practices, the local leader stood out as having the smallest percentage of respondents perceiving that they would provide that survivor-centered practice. The qualitative data bear out, with the following quote exemplifying the diminishing role of the local leader, especially among the youth respondents. “*In the past all the decisions were made by the leader representative of the village and everyone used to trust their decision. But now there are Police and Mayor and Chief of ward. So people take these issues to them*” (Rupandehi, Female youth)*.*

**Table 5:** Perceptions of S&J providers, by respondent group, and sub-sample.

	Total (N = 607)	Adult men (N = 221)		Male youth (N = 75)		Adult women (N = 243)		Female youth (N = 68)	
	% Yes	% Yes	% Don’t Know	% Yes	% Don’t Know	% Yes	% Don’t Know	% Yes	% Don’t Know
Police are trustworthy	84	80	4	89	3	84	5	88	1
Citizens have role in law and order	92	91	3	92	3	93	5	93	1
**Police**									
Willing to report	86	89	0	89	0	83	1	88	0
Case taken seriously	79	79	2	81	7	78	5	84	6
Survivor treated with respect	80	80	2	84	7	76	6	88	6
Privacy protected	75	77	2	79	8	69	8	85	4
Perpetrator punished	77	77	4	82	1	72	9	87	9
Negative repercussions for reporting	59	61	1	60	3	56	6	63	7
**Judicial Committee**									
Willing to report	77	82	2	76	4	75	4	72	9
Case taken seriously	78	82	3	77	5	76	9	74	12
Survivor treated with respect	77	81	4	79	5	74	9	72	15
Privacy protected	72	77	4	75	5	69	11	63	16
Perpetrator punished	72	77	5	72	6	67	13	69	16
Negative repercussions for reporting	58	59	3	63	3	57	7	54	16
**Ward Chairperson**									
Willing to report	80	82	1	79	3	77	2	85	0
Case taken seriously	81	82	2	84	1	79	3	78	9
Survivor treated with respect	80	82	3	82	1	77	4	82	9
Privacy protected	74	77	4	81	3	71	7	71	9
Perpetrator punished	74	77	5	81	1	68	8	77	9
Negative repercussions for reporting	59	62	3	59	1	57	4	60	9
**Local Leader**									
Willing to report	73	77	2	69	7	70	6	71	6
Case taken seriously	73	77	4	71	8	72	9	69	12
Survivor treated with respect	72	76	5	75	8	69	9	71	12
Privacy protected	67	70	5	64	8	63	11	69	12
Perpetrator punished	66	72	6	63	9	63	14	66	13
Negative repercussions for reporting	56	56	3	56	3	56	7	56	10
**Women’s Committee**									
Willing to report	75	73	2	75	3	76	3	75	3
Case taken seriously	78	77	5	76	1	79	5	78	7
Survivor treated with respect	77	75	5	80	1	79	6	77	7
Privacy protected	72	71	5	73	4	73	7	74	7
Perpetrator punished	70	67	8	77	4	70	9	68	10
Negative repercussions for reporting	58	56	4	65	3	58	7	56	10

Out of all age-gender combinations in the sub-study, male youth were most likely to say police were trustworthy. Between 70% and 90% of sub-study participants reported willingness to report physical or sexual victimization to an S&J provider. Men and boys were more willing to report to providers than women and girls, with the exception of the police. The majority of participants stated willingness to report to the police, compared to any other provider, with 90% of all gender and age groups, except women (at 80%), willing to report physical or sexual abuse. Provider types received varying endorsement from participants based on perceptions of how seriously a case would be taken. Eighty percent of men and boys believed that police would protect their female relative’s privacy compared to 70% of adult women. For the other provider types, perceptions that privacy would be maintained were reported by 60% to 80% of participants, with higher proportions of boys, and to a lesser extent men, reporting favorable perceptions of most provider types. Sixty to ninety percent of participants reported a belief that the perpetrator would be punished by S&J providers. Again, police received the highest endorsement, especially among youth, and higher proportions of men and boys reported that they believed the perpetrator would be punished for most provider types. Women and female adolescents are more likely to report “don’t know,” indicating a lack of knowledge about the S&J actor or potentially a gendered fear of speaking out.

### Barriers to GBV help-seeking exist at multiple levels

While there is clear evidence quantitatively – and some evidence qualitatively, especially among youth – that the police and other S&J providers are acceptable to approach, problems remain in availability, accessibility, speed and coordination, corruption, and perceived discrimination on the basis of socioeconomic status and caste as noted in the qualitative interviews. Further, the lack of female S&J providers and female representation, especially within community-focused remediation, remains a challenge to help-seeking. S&J providers identified the community’s lack of awareness as a major barrier to demand, which places survivors at risk of re-traumatization by seeking help from members of the community instead of the police, with out-of-school girls identified as a specifically vulnerable group. S&J provider respondents frequently reported prioritizing community outreach and education as a means of increasing reporting. Stakeholders also suggested that more specifically targeted training of professionals and key community leaders would improve service provision and referral. Diverse stakeholders, including teachers, community members, and S&J providers, emphasized the importance of raising awareness of women’s and children’s rights and of resources available to provide support and assistance to victims of GBV.

*“If a woman gets raped, she goes to five different places for help because of not knowing where to ask help from. Since her parents are uneducated, the child is also uneducated…she would first tell her parents then the parents would tell some of the influential persons of the village. They won’t directly come to the police station. That is the first time she gets raped. Then they would go to the ward chairperson which is the second time she gets raped. After that, they go to villagers and told them about being raped. It is like being raped three different times.” (Kapilvastu, Women’s cell)*

Across qualitative participants, several other challenges were noted. In some cases, community members were aware of S&J services for victims of GBV but were unable to fully access them due to distance and communication barriers. For example, a police officer from Rupandehi cited a lack of local language fluency among staff as a challenge to awareness-raising activities. Participants also stated that while there are police in every community, other organizations are prohibitively far from their communities. S&J providers cited distance and time spent travelling as barriers to victims who wish to escalate complaints within the judicial system. S&J providers also frequently mentioned physical and human resources limitations as barriers to their capacity to address GBV in their communities, with the most commonly reported resource shortage being lack of staff. In terms of specific resources, a few S&J providers cited the need for safehouses to better protect victims and the need for private rooms for questioning at the police station. Additional budget to conduct trainings was also frequently mentioned.

According to the community members who were interviewed, the most frequently reported reason for negative opinions of S&J providers was due to perceptions of bribery, political interference, and discrimination, although it was often unclear whether such perceptions were based on lived experience. For example, one husband in Kapilvastu claimed, “*If you have money, then only you will get help; otherwise, you will not.*” However, help-seekers echoed these sentiments from their own experiences, with three out of four stating that they had faced discrimination based on gender and socioeconomic status.

*“If the decision-maker is a male, all the judgments they make are focused on them and woman are deprived from proper judgment. Since we are female, they won’t listen to us. If a male walks into a decision-making panel, he won’t listen to the words of the women.” (Kapilvastu, Help-seeker)*

S&J providers also cited interference as common reasons for client complaints. In such cases, a perpetrator’s family may advocate on his behalf or involve influential others in the community to persuade the police to ignore the survivor’s complaint. “…*Sometimes, while resolving disputes and conflicts, we are pressurized by political parties or by other people too.”* (S&J provider, Siraha). S&J providers framed it as a hindrance to the execution of their duties that reduced public trust. Concomitantly, participants of all types across all districts cited it as a deterrence to reporting.

### Social and gender norms were perceived to be the most important barrier to help-seeking

**Table 6** describes the norms organized into meta norms, i.e., norms that “[c]onnect with deeply rooted determinants, operate at a more profound level of society, and influence multiple behaviors.” [33] (p.74). Meta norms categories that have been shown to be common to IPV and a range of other behaviors include: authority, privacy, control and violence, gender ideology, protection and social status [33]. From our qualitative findings, we have added meta norms pertaining to harmony and honor as these were common aspects of the norms described.

**Table 6:** Social norms associated with help-seeking, by meta norm, qualitative respondents.

Norm	Meta norm category
Social and familial harmony are more important than individual needs.	Ha; Au
Most problems can and should be handled in the community.	Au
Filial and agnatic loyalty and obedience are expected.	Au; Ss
Elders and men in the community are the decision-makers and others should defer to their opinion.	Au; Gi; Ss
Family members must uphold family honor, especially women.	Ho; Gi
Family matters should not be discussed with others to avoid dishonoring the family.	Ho; Pr
Marriage is for a lifetime and divorce is shameful.	Ho; Gi
It is shameful if families cannot marry their daughters.	Ho; Gi
Violence, unless serious, is considered a normal family occurrence.	Cv
Disobedience is a threat to family harmony and reputation and therefore may justify violence	Ha; Ho; Cv
Women have lower status and are perceived to have fewer capabilities compared to men.	Gi; Ss
Women should be protected from situations that can damage their or their families’ reputation.	Ho; Gi; Po
“Good” or “respectable” women are quiet, do their duties, do not move around unnecessarily, and do not jeopardize family honor.	Ho; Gi;
Women’s sexuality is risky if not controlled through marriage and mobility restrictions.	Gi; Po

**Notes:** Ha: Harmony; Ho: Honor; Au: Authority Pr: Privacy; Cv: Control and violence: Gi: Gender ideology; Po: Protection; Ss: Social status

Qualitatively, a clear social order was described with household members, especially women deferring to men, and both men and women deferring to their elders, who are respected within the household and society. Despite younger generations being more educated than their parents and gaining more decision-making power, deference to men and elders remains the expectation. Participants also indicated that even though girls are now more educated than in the past, they are still raised to believe that the world is not a safe place for them and that they do not have the capability to speak or debate. This belief was also prevalent among men; as described by an S&J provider in Rupandehi, “*almost 75% of males of this society think as such.*” Women are socialized into a cultural model of inequality and are therefore considerably less powerful than men in the social hierarchy. Female qualitative respondents frequently described limitations to their voice and mobility.

*“It is a matter of fear to the women. Males are fearless but female has to fear. The males are able to understand and are aware and so they are fearless and are able to go out of their houses even at nighttime. But women are not able to do such things and it has never happened in our community as well.” (Saptari, Wife)*

It is considered uncommon for wives to challenge or raise their voice or hands against their husbands. Doing so is a violation of gender expectations and roles, widely perceived to be unacceptable and a trigger for violence. When responding to the vignette about a woman who raised her voice against her husband’s unreasonable behavior, most participants responded similarly to a school management committee member stating, “*she did not obey the rules imposed by society; she spoke loudly, she retorted. If one doesn’t act according to the rules of society, violence happens. This is what happens in our society in the Terai*.” *(Siraha, School management committee)*

Qualitative participants explained that because wives join their husbands’ households and become material and social assets for their marital families, they may lack support systems in their nuptial homes. While it is common that a woman returns to her natal family for support or refuge, the duration of refuge and the amount of support she receives is informed by how likely the woman and her family are to face social sanctions in the form of rumors and suspicions that the woman’s marriage is under threat. Divorce is financially untenable for most women, extremely shameful for her entire family, and perceived to be an unnatural state for women. Families have to be careful not to raise suspicions that a woman’s visit to her natal home has the potential to become permanent. These restrictions were reported to be especially strong in the Terai compared to hilly communities. “*If there’s some kind of argument with the husband then she will go to her mother’s house but eventually return to the husband’s house as it looks bad in the society*” *(Siraha, Male youth).*

It is expected that most women will remain married, even after seeking help. Therefore, families face pressure to assist in a reconciliation or else risk having to provide lifelong refuge to their daughter and to live with the humiliation of this breakdown in expected social structure. Most participants reported that families will insist that their daughter not bring the shame and humiliation home with her. “*What will the community say about this and what will they think? Her parents aren’t well off and they won’t accept her; they will rather say ‘don’t come to us and don’t humiliate us, go back to your own house’*” *(Siraha, S&J provider).* Given these challenges, especially the lack of familial support, participants widely perceived that most women would remain silent and bear the burden of violence.

While violence in general was very broadly defined and uniformly considered inappropriate across all sites, violence within the home was not considered a crime, especially when used to correct misbehavior. “*If [women and children] commit mistakes, punishing is not counted as an offense” (Rupandehi, Wife).* Husbands, heads of household, and teachers had the authority and responsibility to discipline errant behavior to maintain order. Our quantitative findings concur as sizable proportions of respondents believed that the use of physical violence by teachers against children, by parents against children, and by husbands against wives was acceptable within their community, especially in Madhesh Province (**Table 7**).

**Table 7:** Mean percent reporting that a majority of their community holds these beliefs, by province, household survey.

Injunctive norms	Madhesh province (N = 1577, 7 sites)	Lumbini province (N = 2253, 10 sites)
Husbands may use force to reprimand their wives because men should be in control of their families	40	28
A woman who complains about her husband’s violent behavior is considered a disloyal wife by her in-laws	38	24
A woman who does not tolerate violence from her husband is dishonoring her family and should not be welcomed home	43	22
A woman who seeks help from the police for domestic violence brings shame on her family and should not be welcomed home	37	25
A person who intervenes when a woman is being beaten by her husband would be considered to be interfering in the couple’s private affairs	41	25
Mediation is the best solution for families who experience domestic violence	45	32
A woman should tolerate violence to keep her family together	41	32
There are times when teachers and school administrators need to use physical force to maintain discipline and order at school	51	27
Women’s groups who get involved in a case of domestic violence usually make the situation worse	38	19
Men should seek the advice of community leaders before allowing a female family member to seek help from a security and justice provider (e.g., police, judicial committee, GBV control group, mediator, mother’s committee)	60	44
Child rearing sometimes requires the use of physical force to discipline children	52	28
Teachers and school administrators should not interfere in a family’s choice of discipline for their children, even if the parents use physical force	51	24
Girls who marry before age 20 will attract a more suitable groom	38	23
Families who do not place appropriate restrictions on their daughter during menstruation deserve the consequences that come with the girl’s impurity	40	23
Marrying a girl soon after the start of her menses will protect her from sexual violence	33	12

Note: 99.7% of the respondents are women.

Qualitatively, respondents were consistent that problems within the family must be solved at home. Only after working without resolution with the family first and the larger community next should the justice sector be approached – that, too, with the support of the family and elders. Forty-four to 60% of survey respondents reported that a majority of their community believed that men should seek advice from community leaders before letting their female family member seek help from S&J providers (**Table 7**), which situates men as the gatekeepers for help-seeking. Women who reported violence to the police without first consulting family members and community leaders would risk losing community support and might incur blame for the violence, according to the qualitative respondents. Across all respondent types, participants consistently believed that GBV could and should be dealt with within the community. Even most of the police qualitatively interviewed indicated that after a report of domestic violence, they would work with the community to come to a solution. Therefore, it was nearly impossible to bypass the community even if one desired to do so. Our quantitative findings (**Table 7**) confirmed that 32% to 45% of participants perceive a community norm that mediation is the best solution for GBV.

### The socially approved approach to dealing with GBV, usually IPV, is some form of compromise and reconciliation even when seeking help from the police

Reconciliation supports the notion of family and community harmony, often at the expense of the victim. While violence clearly detracts from family harmony, violating the social order risks even greater discord. Maintaining the established social order requires the cooperation of all families. Therefore, violating social norms could bring significant negative sanctions, ranging from backbiting to rumors and ostracizing.

*“They [IPV survivors] are afraid of damaging their reputation in the neighborhood. They don’t want the news about the husband beating her to flash out in the community as they are newly married.” (Kapilvastu, Female youth)*

The quantitative data confirm that most people are sensitive to these sanctions and are disinclined to violate them. Between 56% and 59% of surveyed sub-study respondents reported that there would be negative repercussions for reporting, suggesting no real difference in perceived risk of negative consequences of seeking help across provider types (**Table 5**). From the qualitative results, parents and community leaders were reported to be the enforcers of norms against reporting by encouraging women and girls to tolerate violence to avoid shame and humiliation, which extends to the family and often to the entire village. Key reference groups, such as community members and leaders, often pressure women to reconcile with abusive husbands privately rather than involving police or other S&J providers.

*“They used to say I am a bad woman. They taught my husband to kick me out of the house because I used to go to the police and the court to seek justice. They even used to say that I would never go to them for help if I was a good person.” (Rupandehi, Help-seeker)*

While the underlying norms were rather consistent across sites, there were also signs of differences in sensitivity to the sanctions**.** In all sites, while negative reactions to help-seeking for GBV from the community was the norm, there was discussion that some community members might react differently, especially if they are educated. Educated, “aware,” or “understanding” families were reported to be less sensitive to the sanctions associated with the norms and might have a more liberal approach to the problem. Across wards and participants, education was cited as a major factor increasing the likelihood of filing a formal report. Participants of all types stated that educated girls and women are more likely to report and that educated community members, including teachers and family members, would be more supportive of women and girls reporting GBV. This was explained by the greater knowledge and agency that educated individuals have, as well as weaker social norms against reporting among the educated.

*“Those who are less aware think that she has damaged the reputation of not only the family but of the whole village by reporting it to the Police. Those who are aware think that it will bring positive change and will help protect other girls to fall as a victim of such incident.” (Saptari, S&J provider)*

Hilly communities were also recognized as having more liberal norms. As a father-in-law noted, changes are underway regarding women’s rights, which are perceived to be expanding in some settings.

The quantitative data mostly concur. Across the items inquiring about perceptions of S&J actors (**Table 5**), individuals with higher education (upper secondary school or higher) had similar or more positive impressions of the trustworthiness of the police and the degree to which S&J providers would deliver survivor-centered services (**Table 8**). The two exceptions to this pattern included the smaller percentage of persons of higher education who thought that citizens have a role to play in law and order (86% vs. 93%) and the higher percentage of persons of higher education who thought that there would be negative repercussions for reporting to various S&J providers, most pronounced for reporting to the police and the Judicial Committee indicating expectations of some sort of negative social repercussions. Actual experience of repercussions cannot be determined given the very small number of survivors identified in the sub-study who sought help from a S&J provider and survivors who had been recruited on their basis of their help-seeking were not asked these questions as they are hypothetical.

**Table 8:** Perceptions of S&J providers, by education, sub-sample.

Perception	10th grade or lower (N = 499)			11th grade or higher (N = 108)		
	No	Yes	Don’t know	No	Yes	Don’t know
Police are trustworthy	12	84	4	14	82	5
Citizens have role in law and order	4	93	3	10	86	4
**Police**						
Willing to report	12	87	1	18	82	0
Case taken seriously	16	80	4	19	77	5
Survivor treated with respect	15	80	5	18	79	4
Privacy protected	20	74	6	19	78	3
Perpetrator punished	17	77	6	18	78	5
Negative repercussions for reporting	39	56	5	27	71	2
**Judicial Committee**						
Willing to report	19	77	4	18	81	2
Case taken seriously	15	77	7	17	80	4
Survivor treated with respect	16	76	8	14	82	4
Privacy protected	20	71	9	20	76	4
Perpetrator punished	18	71	11	20	18	10
Negative repercussions for reporting	37	57	7	30	67	4
**Ward Chairperson**						
Willing to report	19	80	2	19	82	0
Case taken seriously	16	81	4	18	81	2
Survivor treated with respect	16	80	4	19	80	2
Privacy protected	20	74	6	23	75	2
Perpetrator punished	19	74	7	21	75	4
Negative repercussions for reporting	38	58	4	34	64	2
**Local Leader**						
Willing to report	22	72	6	24	76	0
Case taken seriously	19	73	8	23	75	2
Survivor treated with respect	19	72	9	22	76	2
Privacy protected	23	67	10	31	67	3
Perpetrator punished	22	66	12	27	68	6
Negative repercussions for reporting	40	55	6	35	63	2
**Women’s Committee**						
Willing to report	23	75	3	25	75	0
Case taken seriously	17	78	5	20	78	2
Survivor treated with respect	17	77	6	19	79	8
Privacy protected	21	72	7	24	75	3
Perpetrator punished	22	69	9	24	74	2
Negative repercussions for reporting	37	57	7	34	65	1

Consistent with the qualitative findings, persons of higher education reported that fewer persons in their communities believed the statement, suggesting that their communities, however they defined their community, were less permissive of violence and more accepting of help-seeking (**Table 9**).

**Table 9:** Mean response to the injunctive norm item, by education, household survey.

Injunctive norms	10 grade or lower (N = 3630)		11th grade or higher (N = 180)	
	Mean	SD	Mean	SD
Husbands may use force to reprimand their wives because men should be in control of their families	3	1	4	1
A woman who complains about her husband’s violent behavior is considered a disloyal wife by her in-laws	3	1	4	1
A woman who does not tolerate violence from her husband is dishonoring her family and should not be welcomed home	3	1	4	1
A woman who seeks help from the police for domestic violence brings shame on her family and should not be welcomed home	3	1	4	1
A person who intervenes when a woman is being beaten by her husband would be considered to be interfering in the couple’s private affairs	3	1	3	1
Mediation is the best solution for families who experience domestic violence	3	1	3	1
A woman should tolerate violence to keep her family together	3	1	3	1
There are times when teachers and school administrators need to use physical force to maintain discipline and order at school	3	1	3	1
Women’s groups who get involved in a case of domestic violence usually make the situation worse	3	1	4	1
Men should seek the advice of community leaders before allowing a female family member to seek help from a security and justice provider (e.g., police, judicial committee, GBV control group, mediator, mother’s committee)	3	1	3	1
Child rearing sometimes requires the use of physical force to discipline children	3	1	3	1
Teachers and school administrators should not interfere in a family’s choice of discipline for their children, even if the parents use physical force	3	1	3	1
Girls who marry before age 20 will attract a more suitable groom	3	1	4	1
Families who do not place appropriate restrictions on their daughter during menstruation deserve the consequences that come with the girl’s impurity	3	1	3	1
Marrying a girl soon after the start of her menses will protect her from sexual violence	4	1	4	1

Note: 99.7% of the respondents are women. Higher scores mean fewer members of the respondent’s community believes that statement. 1 = Nearly all; 2 = Most; 3 = Some; 4 = Very few; 5 = None at all

## Discussion

Study findings, contextualized and situated within the literature below, make a significant contribution to the field’s understanding of GBV-related social norms, help-seeking, and security and justice (S&J) service provision in the Madhesh and Lumbini provinces in Nepal. No comparable assessment has been made, in Nepal or elsewhere, across the number of sites and the number of domains of inquiry examined. Our findings highlight the complexity and inter-relatedness of the domains, and provide actionable information for programming in Nepal, and potentially other countries where norms interventions are being deployed alongside more traditional S&J capacity strengthening.

### Descriptive norms

GBV was perceived to be widespread, especially child marriage, IPV, dowry-related domestic violence, and eve-teasing. The higher percentage of persons perceiving forms of GBV to be more widespread in Madhesh province than in Lumbini province is in alignment with existing research on prevalence differences between these two provinces [4]. The perception that GBV is widespread – especially when visible – contributes to its normalization, as has been shown in prior research in Nepal [34,35]. Enhancing the visibility of sanctions, including instances of community members publicly intervening, may help to shift expectations about its acceptability and normality.

### Disclosure

The relatively infrequent reports of IPV in the sub-study are somewhat consistent with, but lower than IPV prevalence estimates generated within the broader study locality in prior IP-SSJ research [36]. The low prevalence of IPV in the sub-study suggests that it may be under-reported, possibly due to social desirability. However, comparable other data at this geographic level is lacking to be able to confirm if the reported estimate is an underestimate. The prevalence of child maltreatment in prior 12 months also does not have a direct comparison. It has been estimated that harsh physical punishment affects at least one in two children according to a nationally representative sample, suggesting that this study’s estimate that approximately 15% of the youth experienced child maltreatment (assessed as emotional, physical, and sexual violence, emotional and physical neglect, and witnessing IPV) is plausible [37].

### Police and help-seeking from S&J providers

In alignment with prior research, respondents preferred community-based reconciliation to resolve issues of GBV [10–13,38]. The emphasis on reconciliation and prioritization of collective needs, which potentially protects social harmony and minimizes threats to family honor, can disadvantage female victims [39]. There is clear recognition across qualitative respondents that escalation of the issue to formal S&J providers is needed, especially when community-based solutions are ineffective, and the violence is repetitive and severe [39]. S&J utilization in situations of severe violence is much more widely perceived to be acceptable in Nepal and elsewhere.

### Challenges to effective S&J service provision

There is some evidence that the police and other S&J providers are becoming more acceptable to approach and that community members perceive that victims will receive gender-sensitive and victim-centered services. Nevertheless, S&J service providers, GBV victims, and other respondents reported continued challenges with local S&J services. Similar findings from previous reports on IP-SSJ study areas suggest that perceptions of corruption and discrimination, interference, lack of female S&J officers, and accessibility are ongoing barriers to formal help-seeking [10–13] that warrant continued redress if efforts to improve S&J acceptability and use for GBV are to be effective.

### Injunctive norms were perceived to be among the most important barriers to help-seeking

In a patriarchal, collectivist society, there are strong expectations of agnatic and filial loyalty and deference. Extended-family living and enduring financial support and care of elders is predominant and has minimally changed over time in Nepal. Evidence from this study suggests that there are some cracks in norms supporting elder decision-making and deference to community mediation as the current generation of adults is more educated than the previous one. Nevertheless, the expectation remains that help-seeking should start with the family and the community, and only when those interventions are ineffective should the issue be escalated to formal S&J services. In this study, it was widely expected that men should seek advice from family and community elders before assisting a female family member to seek justice. Prior research has examined this expectation, showing that while men seek counsel for conflict resolution, they may make their own decision, whereas women are much more tightly bound to the decisions made by men and community elders. Men’s ability to make their own choices aligns with the qualitative findings of this study suggesting that while advice-seeking for conflict resolution is expected for men, they have the agency to decide their course of action whereas women do not.

Perceptions of potential repercussions (negative sanctions) for formal help-seeking were widespread. Help-seeking outside the family and community is perceived to bring dishonor on the husband and family, which, as shown in prior research in Nepal, is a violation of a woman’s familial duty. Further, bypassing family and community mediation and reconciliation can result in strong social sanctions, increased blame placed on women, and a reduction in future social support. Highlighting successful survivor experiences, addressing the S&J service challenges noted above, and working closely with community leaders and families to minimize the social consequences of help-seeking may collectively support a shift in expectations of social sanctions for help-seeking.

Respondents reported that violence was generally unacceptable and a threat to family harmony; however, respondents reported that violence used to correct disobedience, which threatens family order, harmony, and reputation was more acceptable – a reoccurring finding in prior research in Nepal [21,27,40] and globally [41]. The salience of family honor throughout the study’s findings makes it an important norm to link to violence prevention and the acceptability of help-seeking. Respondents perceived that while the perpetrator might be punished, or at least should be punished, women will still be blamed and dishonor will fall to her family. These linkages are not easy to disentangle and require community-based deliberation and stakeholder buy-in to shift the burden of dishonor from the victim to the perpetrator and to allow families to retain their honor by supporting female family members to live free of violence and seek formal justice when needed. While this outward-oriented behavior violates current practices of protecting family honor with secrecy, if reframed as a way to maintain family honor and harmony – values on which there is consensus – help-seeking and providing support for help-seeking might become more acceptable over time.

Girls and boys have access to educational opportunities that were not available to their parents and grandparents. Respondents believed that very early marriage is now generally considered to be harmful to girls and their well-being. However, boys and girls are still socialized into traditional gender roles [42], with the expectation that men will protect and provide for their family and their parents, and women will take care of the home, her husband’s family, bear and raise children, and remain within the home unless necessary. Greater support for the development of women’s and girls’ capabilities to counteract socialization into an inferior, dependent social status is needed to arm them with the skills they need to recognize and secure their rights concomitant with strong male youth engagement to collectively transform gender norms [12,40,43]. In addition to families as the crucible of girls’ and boys’ socialization, schools are an important venue for capability investment and norms change, especially as teachers and other role models have been recognized as change agents for girls’ rights. Engagement of female police officers and other female S&J actors in school, family, and community settings could provide additional, accessible role models for girls.

### Limitations

The study findings must be considered in light of their limitations. The household survey was completed predominantly by married women, limiting assessment of norms’ perceptions to other socio-demographic groups. The sub-study represents only one site among 17 due to budget restrictions, and the study was conducted among sites that were chosen for programming, limiting the generalizability of study findings. However, the household survey and the qualitative data collection were very widespread geographically, underpinning the value of the study’s results. Because of the self-report nature of the data collection and the sensitivity of some questions, it is possible that the data may be biased due to social desirability.

## Conclusion

There is growth in awareness and perceived acceptability of formal S&J providers, although preferences for family- and community-based mediation remain strong. With greater education, there is some evidence that deference to elders may be waning. Ensuring strong family-, school-, and community-based programming will bolster the project’s ability to detect normative and behavior change over its course, doubly so given its simultaneous focus on S&J social accountability and norms, which have been identified in prior IP-SSJ programming to be synergistic catalysts for change [44].

## Supporting information

S1 Table Descriptive and injunctive norms, province mean, tole minimum and maximum. (DOCX)
